# Polymorphisms of long non-coding RNA HOTAIR with breast cancer susceptibility and clinical outcomes for a southeast Chinese Han population

**DOI:** 10.18632/oncotarget.23343

**Published:** 2017-12-16

**Authors:** Yuxiang Lin, Wenhui Guo, Neng Li, Fangmeng Fu, Songping Lin, Chuan Wang

**Affiliations:** ^1^ Department of Breast Surgery, Affiliated Union Hospital of Fujian Medical University, Fuzhou, Fujian Province 350001, China; ^2^ Department of Pathogeny Microbilogy, School of Basic Medical Sciences, Fujian Medical University, Fuzhou, Fujian Province 350108, China; ^3^ Key Laboratory of Tumor Microbiology, School of Basic Medical Sciences, Fujian Medical University, Fuzhou, Fujian Province 350108, China

**Keywords:** breast cancer, HOTAIR, polymorphisms, genetic susceptibility, prognosis

## Abstract

Hox transcript antisense intergenic RNA (HOTAIR) is a well-known long non-coding RNA (lncRNA) which participates in tumorigenesis and progress of multiple cancers. However, the associations among polymorphisms on HOTAIR, breast cancer (BC) susceptibility and clinical outcomes have remained obscure. In this case-control study, we assessed the interaction between three lncRNA HOTAIR single nucleotide polymorphisms (SNPs) (rs1899663, rs4759314 and rs7958904) on the risk and clinical outcome of breast cancer in a Chinese Han population. In total, 969 breast cancer cases and 970 healthy controls were enrolled in this study. Associations among genotypes, BC risk and survival were evaluated by univariate and multivariate logistic regression to estimate the odds ratio (OR), hazard ratio (HR) and its 95% confidence interval (CI). The disease-free survival (DFS) and overall survival (OS) was calculated by the Kaplan–Meier method. We found that the T allele of rs1899663 and C allele of rs7958904 both achieved significant differences between cases and controls in the single locus analyses (*P* = 0.017 and 0.010, respectively). Multivariate analyses also revealed the rs1899663 TT genotype and rs7958904 CC genotype were both at higher risk of breast cancer compared with the GG homozygotes (OR = 2.08, 95% CI = 1.20–3.60 and OR = 1.45, 95% CI = 1.01–2.08, respectively). In survival analysis, we observed that the T allele of rs1899663 presented significant differences for both DFS (HR = 1.64, 95% CI = 1.12–2.40) and OS (HR = 2.10, 95% CI = 1.29–3.42) in younger subjects (age ≤ 40). Our findings may provide new insights into the associations among the genetic susceptibility, the fine classifications and the prognosis of breast cancer. Further studies with larger sample size and functional research should also be conducted to validate our findings and better elucidate the underlying biological mechanisms.

## INTRODUCTION

Worldwide, breast cancer (BC) is one of the most commonly diagnosed malignancies and the primary cause of death from cancer in women [1]. For the year 2017, it is estimated in the United States that approximately 252,710 female patients would be diagnosed with breast cancer and 40,610 would die from it [2]. During the past few decades in China, the incidence of BC has increased rapidly and become the most frequent cancer for women in major cities [3, 4]. The development of breast cancer is a multifactorial and complex process, involving both environmental and genetic factors. Epidemiology studies have demonstrated that age, obesity, menstrual status, positive family history and previous benign breast disease are correlated with the development of breast cancer [5–10]. Whereas accumulative evidences have revealed that, some genetic variants such as single nucleotide polymorphisms (SNPs) in tumor suppressor genes or oncogenes, could also play a critical role in the genetic susceptibility to breast cancer [11–17]. Although a great proportion of publications have focused on the cancer-related polymorphisms that are located in protein-coding genes, several SNPs located in chromosomal regions which do not encode genes are also indicated to contribute to the risk of different cancers.

In the past few years, one novel kind of non-coding RNAs, long-non coding RNA (lncRNA) has attracted extensive attentions for its wide range and comprehensive regulatory functions in human diseases. LncRNA is a type of RNA transcripts that are longer than 200 nucleotides with no protein-coding capacities [18]. Although lncRNAs were identified to be involved in multiple biological processes [19–22], they were also known to play important roles in tumorigenesis, including transcriptional, post-transcriptional and epigenetic regulation of cancer-associated genes, thereby resulting in the cell progression, migration, invasion and apoptosis [23–25]. As one of these RNAs, lncRNA Hox transcript antisense intergenic RNA (HOTAIR) which is located on chromosome 12q13.13, has been proved to be linked with the development and progression of multiple cancers, such as hepatocellular cancer [26, 27], esophageal cancer [28–30], lung cancer [31–33], gastric cancer [34–37] and breast cancer [38–40]. HOTAIR plays a crucial role in gene regulation by modifying the chromatin structure [41]. The 5′ domain of HOTAIR could bind polycomb repressive complex 2 (PRC2), leading to a histone H3 lysine27 trimethylation (H3K27me3) in the HOXD locus, whereas the 3′ domain connects to the LSD1/CoREST/REST complex with H3 lysine 4 demethylation, together regulating the various downstream genes and promoting cancer cell metastasis [42]. In breast cancer, increasing evidences have suggested that lncRNA HOTAIR is an oncogene which is correlated with the BC carcinogenesis, progression and prognosis. Firstly, aberrant up-regulation of HOTAIR was found in breast cancer tissue or plasma samples compared with normal adjacent non-tumorous tissue or healthy controls [43, 44]. Additionally, this high expression of HOTAIR was also a significant predictor of subsequent metastasis and correlated with a shorter survival time in breast cancer patients [38, 43]. Moreover, *in vitro* studies have identified that the HOTAIR was robustly expressed in the basal-like breast cancer cells and the inhibition of HOTAIR could reduce the basal-like gene expression and growth [45]. Recently, several single nucleotide polymorphisms located in HOTAIR were also reported to show highly significant associations with breast cancer. For example, one study by Yan *et al.* [46] identified that the T allele of rs920778 conferred significant increased risk to BC, with the other study in Turkey indicating that the TT genotype of rs12826786 might play critical roles in genetic susceptibility for breast cancer [47]. However, our understanding for the association between lncRNA HOTAIR polymorphisms and the genetic susceptibility of BC is still at an early stage. And as far as we know, no published studies have ever evaluated the relationships between HOTAIR SNPs and the clinical outcomes in breast cancer patients. Accordingly, we selected five SNPs (rs12826786, rs1899663, rs4759314, rs7958904 and rs920778) which were previously identified to be associated with cancer risk and conducted this present case-control study involving 969 BC patients and 970 healthy controls, aiming to investigate the role of HOTAIR tag SNPs on the risk and clinical outcome of breast cancer in a southeast Chinese Han population.

## RESULTS

### Subject characteristics

A total of 1939 subjects (969 cases and 970 healthy controls) were involved in this study. The selected demographic characteristics and clinicopathological features of breast cancer cases and control subjects are displayed in Table 1. No significant differences were observed between cases and controls in age, menopausal status, age at menopause and previous benign disease (*P >* 0.05). Compared with the healthy controls, the BC patients were more likely to have a lower mean BMI, an earlier age at menarche, a later age at first live birth and a higher proportion of family history of breast cancer (*P* < 0.05). Among 969 breast cancer cases, 584 (60.3%) were with tumor size >2 cm, 385 (39.7%) were with tumor size ≤2 cm, 490 (50.6%) patients had lymph node involvement, 479 (49.4%) patients did not have lymph node involvement. Moreover, 644 (66.4%) cases were luminal type, 149 (15.4%) were HER-2 overexpressing and 176 (18.2%) were triple negative breast cancer (TNBC).

**Table 1:** Basic demographic characteristics and clinical features for breast cancer cases and cancer free-controls

Characteristics	Cases (*n* = 969) no.(%)	Controls (*n* = 970) no.(%)	*P*
Age, y (mean ± SD)	46.9 ± 10.2	47.2 ± 11.0	0.556
BMI, kg/m^2^ (mean ± SD)	22.5 ± 2.6	23.1 ± 3.1	<0.001
Age at menarche, y (mean ± SD)	15.2 ± 1.7	15.5 ± 1.7	<0.001
Menopausal status			0.053
Premenopausal	620	624	
Postmenopausal	342	327	
Unnatural menopause^a^	7	19	
Age at menopause, y (mean ± SD)	50.2 ± 3.0	50.3 ± 3.0	0.673
Age at first live birth, y (mean ± SD)	25.0 ± 3.6	24.2 ± 3.3	<0.001
Family history of breast cancer			<0.001
Yes	75	12	
No	894	958	
Previous benign breast disease			0.079
Yes	40	26	
No	929	944	
Tumor size			
>2 cm	584 (60.3)		
≤2 cm	385 (39.7)		
Lymph node involvement			
Yes	490 (50.6)		
No	479 (49.4)		
Estrogen receptor (ER) status			
Positive	644 (66.5)		
Negative	325 (33.5)		
Progestrone receptor (PR) status			
Positive	566 (58.4)		
Negative	403 (41.6)		
HER-2 status			
Positive	315 (32.5)		
Negative	654 (67.5)		
Molecular subtype			
Luminal type	644 (66.4)		
HER-2 overexpression	149 (15.4)		
TNBC^b^	176 (18.2)		
Relapse			
Yes	337 (34.8)		
No	632 (65.2)		
Death			
Yes	217 (22.4)		
No	752 (77.6)		

^a^Unnatural menopause consists of hysterectomy operation and other status.

^b^TNBC for triple negative breast cancer.

### Effects of HOTAIR SNPs and breast cancer risk

**Figure 1:**
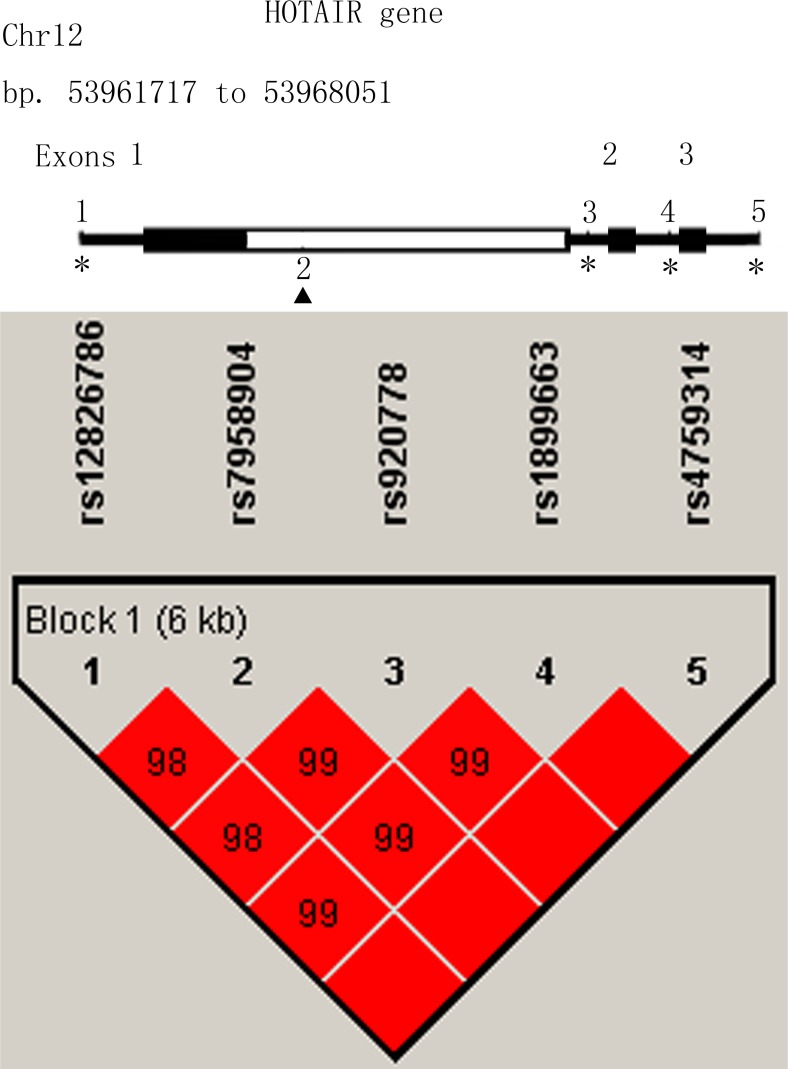
Linkage disequilibrium and genomic location of HOTAIR polymorphisms

In linkage disequilibrium (LD) analysis, the SNP rs12826786 was discovered in strong LD with rs1899663, with a Pearson’s correlation coefficient (*r*^2^) of 0.983. Similarly, the SNP rs920778 was also in strong LD with rs7958904, with a Pearson’s correlation coefficient (*r*^2^) of 0.984 (Figure 1). So rs1899663, rs4759314 and rs7958904 were selected as three tag SNPs in this study. The genotype distributions of all three tag SNPs are shown in Table 2. The observed genotype frequencies in three SNPs were consistent with those expected from Hardy-Weinberg equilibrium (HWE) in healthy controls (*P* = 0.402 for rs1899663, *P* = 0.295 for rs4759314 and *P* = 0.764 for rs7958904, respectively). In the single locus analyses, the T allele of rs1899663 and C allele of rs7958904 both achieved significant differences between cases and controls, with the *P* value of 0.017 and 0.010, respectively. Multivariate logistic regression analyses adjusted by age, BMI, age at menarche, menopausal status and family history of breast cancer also revealed that, for rs1899663 and rs7958904, the TT or CC carriers were both at higher risk of breast cancer compared with the GG homozygotes (OR =  2.08, 95% CI =  1.20–3.60 and OR = 1.45, 95% CI = 1.01–2.08, respectively). However, when we combined the GT and TT genotype of rs1899663, or the GC and CC genotype of rs7958904 to construct a dominant model, no significant increased risk was found. In addition, we didn’t detect any significant correlation for rs4759314 in allelic, co-dominant or dominant model. In the power analysis, we had power of 85.64% and 30.1% to detect an OR of 2.08 (1.20–3.60) and an OR of 1.45 (1.01–2.08) for rs1899663 and rs7958904 for co-dominant model, respectively.

**Table 2:** Distribution of genotype/allele frequency of three SNPs in HOTAIR and their correlations with breast cancer

Genotype	Cases (*n* = 969) no.(%)	Controls (*n* = 970) no.(%)	*P*	Adjusted OR (95% CI)^a^	*P* trend^b^
rs1899663 G>T					
GG	628	665		1.00 (Reference)	
GT	299	284	0.326	1.11 (0.91–1.35)	
TT	42	21	0.009	2.08 (1.20–3.60)	0.027
GT + TT	341	305	0.109	1.17 (0.97–1.42)	
T allele frequency	383 (19.8)	326 (16.8)	0.017^c^		
rs4759314 A>G					
AA	801	817		1.00 (Reference)	
GA	157	144	0.536	1.08 (0.84–1.39)	
GG	11	9	0.805	1.12 (0.45–2.80)	0.520
GA + GG	168	153	0.514	1.08 (0.85–1.39)	
A allele frequency	179 (9.2)	162 (8.4)	0.338^c^		
rs7958904 G>C					
GG	489	537		1.00 (Reference)	
GC	396	373	0.171	1.14 (0.94–1.38)	
CC	84	60	0.046	1.45 (1.01–2.08)	0.030
GC + CC	480	433	0.068	1.19 (0.99–1.42)	
C allele frequency	564 (29.1)	493 (25.4)	0.010^c^		

^a^Adjusted by age, BMI, age at menarche, menopausal status and family history of breast cancer where appropriate.

^b^*P* trend for genotypes between cases and controls.

^c^Two-sided χ^2^ test for differences in allele frequency distributions between cases and cancer-free controls.

### Stratified analysis of HOTAIR polymorphisms and breast cancer

To further assess the suggestive association between HOTAIR polymorphisms and the risk of breast cancer, we conducted stratified analyses among different subgroups of demographic characteristics and reproductive factors in dominant model (Table 3). For the T carriers of rs1899663, elevated risks of BC were found in subgroups of younger patients (age ≤ 40) (OR = 1.48, 95% CI = 1.04–2.12), individuals with earlier menarche (OR = 1.38, 95% CI = 1.05–1.82) and subjects with an earlier age at first live birth (OR = 1.36, 95% CI = 1.06–1.75). As for the C carriers of rs7958904, we observed significantly increased risks in subgroup of lower BMI individuals (BMI ≤ 24) (OR = 1.26, 95% CI = 1.01–1.57), individuals with an earlier age at first live birth (OR = 1.37, 95% CI = 1.08–1.74) and patients with ER positive (OR = 1.32, 95% CI = 1.07–1.61) or PR positive (OR = 1.32, 95% CI = 1.07–1.64). No positive associations were detected in any of the subgroups of rs4759314 (Supplementary Table 1). Also, no significant heterogeneity was discovered within any of the subgroup for the three tag SNPs.

**Table 3:** Stratified analysis on associations among rs1899663 and rs7958904 polymorphisms and breast cancer risk

Characteristics	rs1899663		*P*	OR (95% CI)^a^	*P*^b^	rs7958904		*P*	OR (95% CI)^a^	*P*^b^
	Cases ( GG/GT + TT)	Controls (GG/GT + TT)				Cases (GG/GC + CC)	Controls (GG/GC + CC)			
Age					0.155					0.525
≤40	164/112	192/89	0.031	1.48 (1.04–2.12)		129/147	153/128	0.118	1.31 (0.93–1.85)	
>40	464/229	473/216	0.662	1.05 (0.84–1.33)		360/333	384/305	0.250	1.14 (0.91–1.41)	
BMI					0.793					0.319
≤24	451/250	436/212	0.247	1.15 (0.91–1.44)		343/358	355/293	0.040	1.26 (1.01–1.57)	
>24	177/91	229/93	0.283	1.22 (0.85–1.75)		146/122	182/140	0.868	1.03 (0.73–1.44)	
Age at menarche					0.100					0.503
≤15	314/182	349/149	0.019	1.38 (1.05–1.82)		244/252	274/224	0.074	1.26 (0.98–1.63)	
>15	314/159	316/156	0.902	0.98 (0.74–1.30)		245/228	263/209	0.450	1.11 (0.85–1.44)	
Menopausal status					0.649					0.490
Premenopausal	402/218	426/198	0.261	1.15 (0.90–1.46)		310/310	350/274	0.056	1.25 (0.99–1.57)	
Postmenopausal	220/122	227/100	0.162	1.27 (0.91–1.78)		174/168	175/152	0.605	1.09 (0.79–1.49)	
Age at menopause					0.624					0.434
≤50	87/56	118/49	0.471	1.19 (0.75–1.89)		104/95	88/79	0.862	0.96 (0.63–1.48)	
>50	133/66	109/51	0.163	1.42 (0.87–2.31)		70/73	87/73	0.327	1.26 (0.79–2.03)	
Age at first live birth					0.129					0.057
≤25	329/195	445/198	0.017	1.36 (1.06–1.75)		251/273	362/281	0.010	1.37 (1.08–1.74)	
>25	270/137	191/96	0.930	0.99 (0.71–1.37)		216/191	149/138	0.677	0.94 (0.69–1.28)	
ER status					0.532					0.084
Positive	415/229		0.057	1.23 (0.99–1.53)		313/331		0.009	1.32 (1.07–1.61)	
Negative	213/112		0.480	1.10 (0.84–1.45)		176/149		0.931	0.99 (0.76–1.28)	
PR status					0.923					0.148
Positive	369/197		0.113	1.20 (0.96–1.50)		275/291		0.010	1.32 (1.07–1.64)	
Negative	259/144		0.187	1.18 (0.92–1.52)		214/189		0.724	1.04 (0.82–1.32)	

^a^Adjusted by age, BMI, age at menarche, menopausal status and family history of breast cancer where appropriate.

^b^*P* for heterogeneity test.

### Effects of clinicopathological features and HOTAIR SNPs on breast cancer survival

**Figure 2:**
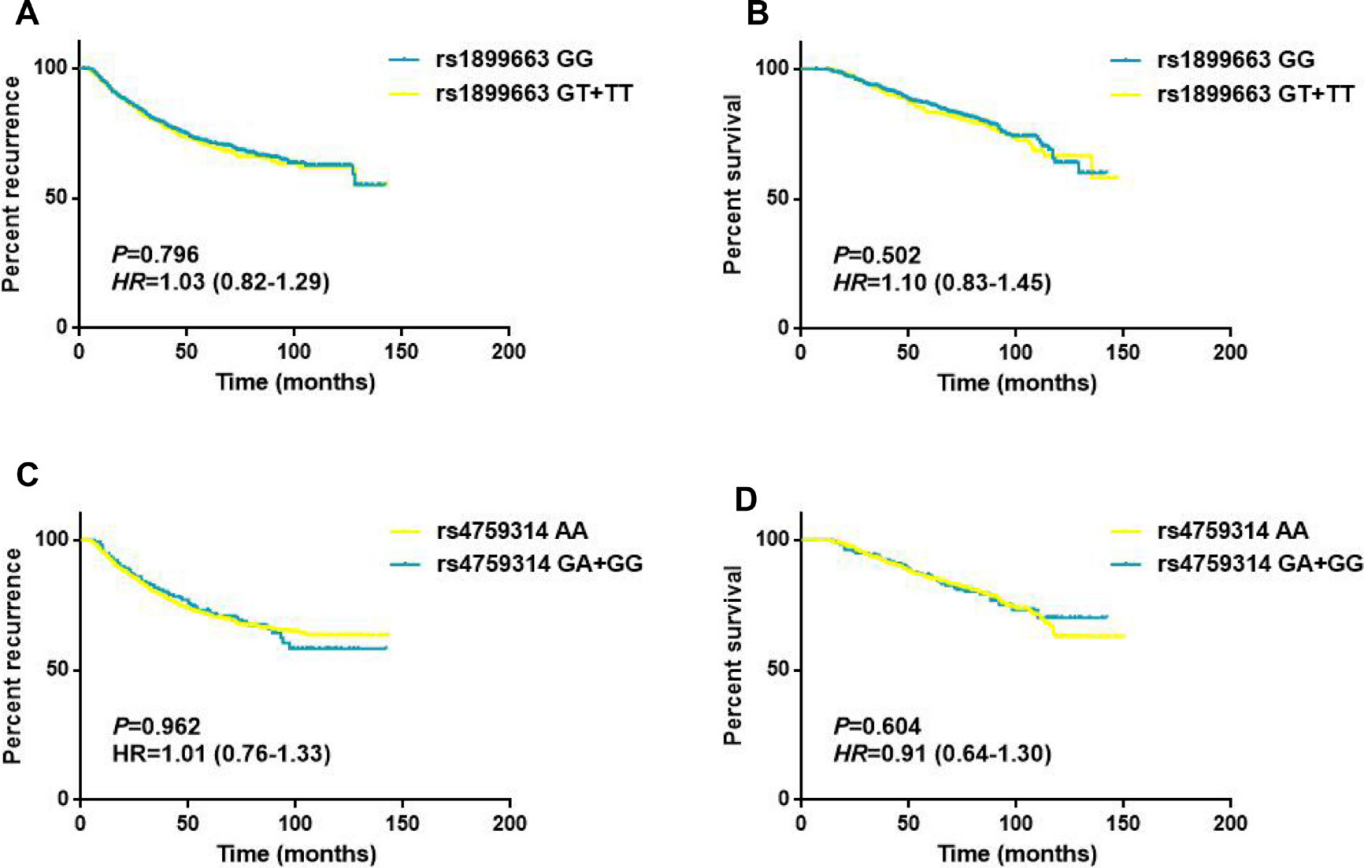
Survival curves for rs1899663 and rs4759314 in total patients (**A**) Disease free survival of the patients grouped according rs1899663 genotypes. (**B**) Overall survival of the patients grouped according rs1899663 genotypes. (**C**) Disease free survival of the patients grouped according rs4759314 genotypes. (**D**) Overall survival of the patients grouped according rs4759314 genotypes.

**Figure 3:**
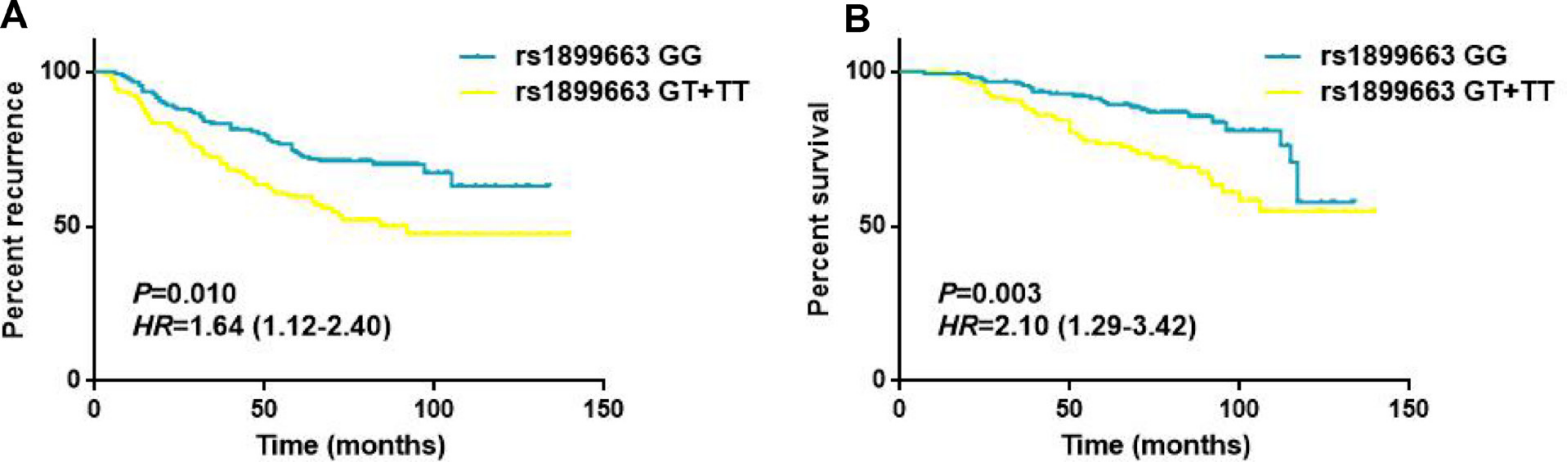
Survival curves for rs1899663 in younger patients (age ≤ 40) subgroup (**A**) Disease free survival of the younger subjects (age ≤ 40) grouped according rs1899663 genotypes. (**B**) Overall survival of the younger subjects (age ≤ 40) grouped according rs1899663 genotypes.

As shown in Table 4, the associations of clinicopathological features and HOTAIR polymorphisms with patients’ disease free survival and overall survival were evaluated by Cox regression analyses. The results demonstrated that tumor size, lymph node involvement and different molecular subtypes were significantly associated with the DFS and OS for breast cancer patients (all *P* < 0.05, log-rank test). While for the HOTAIR tag SNPs, no statistically significant associations were observed between the genotypes and the survival of breast cancer in any of the genetic models (Figure 2). To further assess the prognostic value of HOTAIR polymorphisms, we also performed stratified analyses by age, tumor size, lymph node involvement and different molecular subtypes. Multivariate analyses revealed that the T carriers of rs1899663 presented significant differences for both DFS (HR = 1.64, 95% CI = 1.12–2.40) and OS (HR = 2.10, 95% CI = 1.29–3.42) in younger patients (age ≤ 40) subgroup (Table 5 and Figure 3). As for the G carriers of rs4759314, we observed a decreased risk for OS (HR = 0.26, 95% CI = 0.08–0.83) in patients without lymph node involvement (Table 6). However, we did not notice any significant difference in survival for rs7958904 (Supplementary Table 2) or within any of the other subgroups of rs1899663 and rs4759314.

**Table 4:** Multivariate analysis of prognostic factors with DFS and OS for breast cancer patients

Characteriscs	Disease free survival			Overall survival		
	Patients (Relapse)	HR (95% CI)^a^	Log-rank *p*	Patients (Death)	HR (95% CI)^a^	Log-rank *p*
Age						
>40	693 (229)	1.00		693 (151)	1.00	
≤40	276 (108)	1.17 (0.81–1.67)	0.404	276 (66)	1.16 (0.74–1.81)	0.512
Tumor size						
≤2 cm	385 (81)	1.00		385 (53)	1.00	
>2 cm	584 (256)	2.44 (1.90–3.14)	<0.01	584 (164)	2.24 (1.65–3.06)	<0.01
Lymph node involvement						
No	479 (104)	1.00		479 (50)	1.00	
Yes	490 (233)	2.61 (2.07–3.29)	<0.01	490 (167)	3.81 (2.78–5.23)	<0.01
Molecular subtype						
Luminal type	644 (191)	1.00		644 (109)	1.00	
HER-2 overexpression	149 (65)	1.78 (1.34–2.36)	<0.01	149 (46)	2.16 (1.53–3.05)	<0.01
TNBC	176 (81)	1.89 (1.45–2.45)	<0.01	176 (62)	2.56 (1.87–3.49)	<0.01
Adjuvant chemotherapy						
Yes	919 ( 325)	1.00		919 (211)	1.00	
No	50 (12)	1.46 (0.82–2.61)	0.201	50 (6)	2.12 (0.94–4.82)	0.072
rs1899663 G>T						
GG	628 (217)	1.00		628 (137)	1.00	
GT	299 (102)	0.97 (0.77–1.23)	0.825	299 (69)	1.06 (0.80–1.42)	0.685
TT	42 (18)	1.53 (0.94–2.48)	0.086	42 (11)	1.41 (0.76–2.61)	0.277
GT + TT	341 (120)	1.03 (0.82–1.29)	0.796	341 (80)	1.10 (0.83–1.45)	0.502
rs4759314 A>G						
AA	801 (275)	1.00		801 (179)	1.00	
GA	157 (61)	1.06 (0.80–1.40)	0.669	157 (37)	0.94 (0.66–1.34)	0.734
GG	11 (1)	0.24 (0.03–1.72)	0.156	11 (1)	0.43 (0.06–3.07)	0.400
GA + GG	168 (62)	1.01 (0.76–1.33)	0.962	168 (38)	0.91 (0.64–1.30)	0.604
rs7958904 G>C						
GG	489 (168)	1.00		489 (106)	1.00	
GC	396 (136)	0.96 (0.76–1.20)	0.690	396 (89)	0.99 (0.75–1.31)	0.945
CC	84 (33)	1.20 (0.82–1.74)	0.348	84 (22)	1.25 (0.79–1.98)	0.339
GC + CC	480 (169)	0.99 (0.80–1.23)	0.959	480 (111)	1.03 (0.79–1.34)	0.811

^a^Data were estimated by Cox regression analyses with adjustment for age, tumor size, lymph node status, ER,PR and Her-2 status where appropriate.

**Table 5:** Stratified analysis of HOTAIR rs1899663 genotypes on DFS and OS of breast cancer patients

Variable	Disease free survival Genotypes (Relapse/Patients)					Overall survival Genotypes (Death/Patients)				
	GG	HR (95% CI)	GT + TT	HR (95% CI)^a*^	Log-rank *p*	GG	HR (95% CI)	GT + TT	HR (95% CI)^a*^	Log-rank *p*
Age										
≤40	54/164	1.00	54/112	1.64 (1.12–2.40)	0.010	28/164	1.00	38/112	2.10 (1.29–3.42)	0.003
>40	163/464	1.00	66/229	0.80 (0.60–1.07)	0.130	109/464	1.00	42/229	0.78 (0.54–1.12)	0.175
Tumor size										
≤2 cm	50/244	1.00	31/141	1.09 (0.70–1.71)	0.700	33/244	1.00	20/141	1.05 (0.60–1.83)	0.875
>2 cm	167/384	1.00	89/200	1.05 (0.81–1.36)	0.719	104/384	1.00	60/200	1.14 (0.83–1.57)	0.407
Lymph node involvement										
No	66/322	1.00	38/157	1.21 (0.81–1.80)	0.356	33/322	1.00	17/157	1.09 (0.61–1.95)	0.779
Yes	151/306	1.00	82/184	0.87 (0.66–1.14)	0.304	104/306	1.00	63/184	1.04 (0.76–1.43)	0.807
Molecular subtype										
Luminal type	125/415	1.00	66/229	0.95 (0.70–1.28)	0.727	70/415	1.00	39/229	1.04 (0.70–1.54)	0.861
HER-2 overexpression	41/98	1.00	24/51	1.24 (0.75–2.05)	0.411	29/98	1.00	17/51	1.22 (0.67–2.22)	0.512
TNBC	51/115	1.00	30/61	1.12 (0.71–1.77)	0.616	38/115	1.00	24/61	1.18 (0.70–1.96)	0.536

^a^Cox regression analyses for DFS and OS in breast cancer patients according to dominant model.

^*^Adjusted by age, tumor size, lymph node status, ER,PR and Her-2 status where appropriate.

**Table 6:** Stratified analysis of HOTAIR rs4759314 genotypes on DFS and OS of breast cancer patients

Variable	Disease free survival Genotypes (Relapse/Patients)					Overall survival Genotypes (Death/Patients)				
	AA	HR (95% CI)	GA + GG	HR (95% CI)^a*^	Log-rank *p*	AA	HR (95% CI)	GA + GG	HR (95% CI)^a*^	Log-rank *p*
Age										
≤40	85/223	1.00	23/53	1.06 (0.67–1.68)	0.806	52/223	1.00	14/53	1.03 (0.57–1.87)	0.919
>40	190/578	1.00	39/115	0.97 (0.69–1.37)	0.871	127/578	1.00	24/115	0.86 (0.56–1.33)	0.501
Tumor size										
≤2 cm	65/318	1.00	16/67	1.09 (0.63–1.88)	0.768	46/318	1.00	7/67	0.63 (0.28–1.40)	0.254
>2 cm	210/483	1.00	46/101	0.99 (0.71–1.36)	0.926	133/483	1.00	31/101	1.05 (0.71–1.56)	0.803
Lymph node involvement										
No	90/394	1.00	14/85	0.99 (0.97–1.01)	0.331	47/394	1.00	3/85	0.26 (0.08–0.83)	0.023
Yes	185/407	1.00	48/83	1.29 (0.94–1.78)	0.115	132/407	1.00	35/83	1.20 (0.83–1.76)	0.334
Molecular subtype										
Luminal type	151/521	1.00	40/123	1.05 (0.74–1.49)	0.780	89/521	1.00	20/123	0.85 (0.52–1.38)	0.498
HER-2 overexpression	54/128	1.00	11/21	1.37 (0.71–2.64)	0.347	36/128	1.00	10/21	1.95 (0.96–3.96)	0.064
TNBC	70/152	1.00	11/24	0.89 (0.47–1.69)	0.724	54/152	1.00	8/24	0.77 (0.36–1.63)	0.488

^a^Cox regression analyses for DFS and OS in breast cancer patients according to dominant model.

^*^Adjusted by age, tumor size, lymph node status, ER,PR and Her-2 status where appropriate.

## DISCUSSION

Deeper understanding of lncRNAs and their roles in tumor pathogenesis, progression and prognosis could contribute a large number of potential clues to develop novel therapeutic approaches for breast cancer. HOTAIR (HOX transcript antisense RNA) is known as a functional lncRNA which participates in several tumor types including breast cancer [26–40]. The oncogenic roles of HOTAIR have attracted extensive attentions in breast cancer, while epidemiological studies focusing on tumor susceptibility and prognosis conferred by genetic polymorphisms in its locus have not been widely investigated [38–40, 43]. In this present study, we evaluated the effects of three potential functional HOTAIR polymorphisms (rs1899663, rs4759314 and rs7958904) on breast cancer susceptibility and clinical outcomes in a Chinese population. We identified individuals with T allele of rs1899663 and C allele of rs7958904 had an increased risk of developing breast cancer and patients with T carriers of rs1899663 presented a worse DFS and OS in subgroup with younger subjects. Our findings support the hypothesis that the functional genetic variants located in HOTAIR may explain a part of BC genetic basis. And to the best of our knowledge, this is the first study to evaluate the correlations between HOTAIR variants and breast cancer survival.

LncRNA HOTAIR is located on chromosome 12q13.13 and plays a key role in gene regulation by modifying the chromatin structure [41]. The 5′ domain of HOTAIR could bind polycomb repressive complex 2 (PRC2) and leads to a histone H3 lysine27 trimethylation (H3K27me3) in the HOXD locus, while the 3′ domain connects to the LSD1/CoREST/REST complex with H3 lysine 4 demethylation, together regulating the various downstream genes and promoting cancer cell metastasis [42]. HOTAIR has been widely explored in breast cancer and suggested as a functional lncRNA which is correlated with the carcinogenesis, progression and prognosis of BC. The aberrant up-regulation of HOTAIR was proved to be found in breast cancer tissue or plasma samples compared with the normal adjacent tissue or healthy controls [43, 44], and this high expression was also indicated as a predictor of subsequent metastasis and correlated with a shorter survival time of breast cancer patients [38, 43]. Except from these, HOTAIR was additionally reported to be robustly expressed in the basal-like breast cancer cells and the inhibition of HOTAIR could reduce the basal-like gene expression and growth *in vitro* studies [45]. Therefore, understanding the biological roles of HOTAIR may help us to recruit this lncRNA as a diagnostic or predictive biomarker in breast cancer.

In current study, we demonstrated that the T allelic frequency of rs1899663 and C allelic frequency of rs7958904 were both significantly higher in breast cancer cases compared with the cancer-free controls. Multivariate analyses on genotype distributions also revealed that the TT carriers of rs1899663 and the CC carriers of rs7958904 were consistently associated with the elevated risk of breast cancer. In further stratified analyses, we observed that the T carriers of rs1899663 were correlated with elevated risks of BC in subgroups of younger patients (age ≤ 40), individuals with earlier menarche and subjects with an earlier age at first live birth. As for the C carriers of rs7958904, increased risks of breast cancer were found to be more evident in subgroup of lower BMI individuals (BMI ≤ 24), individuals with an earlier age at first live birth and patients with ER positive or PR positive. These results showed that the effects of HOTAIR genetic variant on breast cancer risk could be modulated by specific environmental exposures as well as demographic factors, and provided evidence supporting that the carcinogenesis is a complex process involving both genetic and environmental factors. Previous studies have suggested that the T allele of rs1899663 was associated with a higher risk of developing prostate cancer [48], whereas this significant positive correlation was not detected in cervical cancer [49] and esophageal squamous cell carcinoma [50]. In one study concerning HOTAIR polymorphisms and breast cancer [51], the rs1899663 T allele also did not show significant differences in the frequency distribution of cancer patients and healthy controls in an overall correlation analysis, while the follow-up stratified analysis indicated the GT+TT genotypes had a significantly lower risk of BC among women with age at menarche >14 (OR = 0.42, 95% CI = 0.21–0.82) and number of pregnancies >2 (OR = 0.65, 95% CI = 0.49–0.95). As for rs7958904, several studies have indicated that the C allele was associated with a significantly decreased risk of colorectal cancer [52], ovarian cancer [53] and osteosarcoma [54] when compared with the G allele, which produce a contrary result with our study. This may be interpreted by the different susceptibilities to a disease among the different populations and the different kinds of cancer could have various etiologies, which involve diverse genetic or epigenetic modifications.

The polymorphism rs1899663 and rs7958904 was separately located on the intron 2 and exon 6 of HOTAIR gene. Guo *et al.* [55] have reported that HOTAIR SNP rs12826786 which is in strong LD with rs1899663 (*r*^2^ = 0.983) was associated with gastric cardia adenocarcinoma risk and had an allelic-specific effect on HOTAIR expression. It is plausible that the rs1899663 or its LD polymorphisms could affect the BC susceptibility by altering the HOTAIR expressions. *In silico* analyses have revealed that, the secondary structure of HOTAIR gene was distinctly changed with the rs7958904 G/C variants, indicating that this polymorphism may participate in tumorigenesis through the alteration of HOTAIR structure [52]. Another explanation for rs7958904 in relation to breast cancer susceptibility is that the real functional SNP is rs920778, which is in high LD (*r*^2^ = 0.984) with rs7958904. Polymorphism rs920788 was also located on the intron of HOTAIR gene and was proved to be able to enhance the intronic enhancer activity and increase HOTAIR expression in several cancer cells [49, 50].

In overall survival study, we did not notice any significant association between genotypes of three tag SNPs and the survival of breast cancer in any of the genetic models. While in the subsequent stratified analysis, we revealed that the T allele of rs1899663 presented significant differences for both DFS (HR = 1.64, 95% CI = 1.12–2.40) and OS (HR = 2.10, 95% CI = 1.29–3.42) in younger subjects (age ≤ 40) and the G allele of rs4759314 showed a decreased risk for OS (HR = 0.26, 95% CI = 0.08–0.83) in patients without lymph node involvement. However, given the small sample of rs4759314 GG carriers in subgroups without lymph node involvement in overall analysis (3 cases), we speculated that the association of rs4759314 observed in OS study may be a false positive result.

In conclusion, we identified two SNPs located in HOTAIR (rs1899663 and rs7958904) that were significantly associated with the increased risk of breast cancer and firstly investigated the role of HOTAIR tag SNPs on the clinical outcome of BC in a southeast Chinese Han population. However, several limitations in this study should also be mentioned. Firstly, the sample size of the current study was still not large enough and might lead to a limited statistical power and impact on the accuracy and precision of the results. Secondly, we only included three lncRNA HOTAIR polymorphisms in the present study, while studies comprising more functional SNPs in HOTAIR might be more able to illuminate the precise role of genetic variants in BC carcinogenesis and progress. Thirdly, the biological function of the HOTAIR polymorphisms is not clear, further functional studies are still needed to explore the relationship. In spite of these limitations, the findings of our study were still informative for the researchers and physicians in this field. Additional prospective population-based studies with larger sample size and different ethnicities, as well as relevant functional studies are still needed to confirm our findings.

## MATERIALS AND METHODS

### Ethical statement

This study and consent procedure was approved by the Ethical Committee of Affiliated Union Hospital of Fujian Medical University. Each participant included in the study has provided a written informed consent document.

### Study subjects

This hospital-based study was conducted on a total of 969 breast cancer patients and 970 healthy free controls. All participants were genetically unrelated Chinese Han residents of Fujian Province and its surrounding regions. Breast cancer subjects were all histopathologically confirmed with primary breast cancer and recruited from the Affiliated Union Hospital of Fujian Medical University between July 1995 and October 2010. Healthy controls (frequency-matched to cases on age ±3 years) were randomly selected from individuals attending routine health examination in the outpatients’ department during the same period. Each patient and healthy control was interviewed face-to-face by two trained oncologists to gather information on demographic factors, menstrual status, fertility status, previous benign breast disease history and the family history of breast cancer. Specific clinicopathological data of breast cancer cases including tumor size, lymph node involvement, estrogen receptor (ER), progesterone receptor (PR) and human epidermal growth factor receptor-2 (HER-2) status were all extracted from medical records and pathology reports. The molecular subtypes of breast cancer based on immunohistochemical (IHC) profiles were categorized as follows: Luminal subtype = ER+ or PR+, and HER2±; HER2 overexpression (HER2+) = ER−, PR−, and HER2+; Triple-negative breast cancer (TNBC) = ER−, PR−, and HER2−.

### Outcome collections

Disease free survival (DFS) and overall survival (OS) were the main study points. Patients alive on the last follow-up date were considered censored. DFS was measured as the time from the date of diagnosis to the first local or distant recurrence or to the last follow-up. OS was defined as the time from the date of diagnosis to the date of death due to all causes (including breast cancer) or the last follow-up. The date of death was obtained from inpatient and outpatient records or by the relatives of patients through follow-up telephone calls. The last follow-up date of this study was November 1st, 2016.

### DNA extraction and genotyping

Each participant was asked to provide a 5-ml peripheral blood sample after enrolling in this study. Genomic DNA was extracted from the peripheral-blood samples using a Whole-Blood DNA Extraction Kit (Bioteke, Beijing, China) following the manufacturer’s instructions. LncRNA HOTAIR tag SNPs were genotyped by a 2 × 48-Plex SNPscan Kit (Cat#:G0104K; Genesky Biotechnologies Inc., Shanghai, China). The DNA samples were ligated and amplified by polymerase chain reaction (PCR) according to the standardization protocol recommended by the manufacturer. Ligation products were performed with an ABI3730XL sequencer and the raw data was analyzed by GeneMapper 4.1 Software (Applied Biosystems, Foster City, CA). For quality control, all genotyping were performed without knowledge of case or control status. About 10% of the DNA samples were randomly selected for direct sequencing (BGI Sequencing, Beijing), and the result was 100% concordant.

### Statistical analysis

All statistical analyses were performed using Statistical Package for the Social Sciences (SPSS, version 21.0) for Windows (SPSS, Chicago, IL). The differences between breast cancer cases and healthy controls in demographic characteristics and environmental risk factors were evaluated by using the Student’s *t*-test (for continuous variables) and chi-squared (χ^2^) test (for categorical variables). Hardy-Weinberg equilibrium (HWE) was applied by a goodness-of-fit chi-squared (χ^2^) test to assess the expected and observed genotype frequencies in control subjects. Associations among genotypes, breast cancer risk and survival were evaluated by the computing odds ratio (OR), hazard ratio (HR) and its 95% confidence interval (CI) from univariate and multivariate logistic regression analyses. Linkage disequilibrium was calculated from genotype data using Haploview 4.1 (http://www.broad.mit.edu/mpg/haploview/). The power analysis of this study was performed by using the QUANTO program, version 1.2.4, with the disease risk for the Chinese population was 268 per 100000. The disease free survival and overall survival was calculated by the Kaplan–Meier method, with the log-rank test used to compare the differences. All statistical analyses were two-sided, and a level of *P* value less than 0.05 was considered significant.

## SUPPLEMENTARY MATERIALS TABLES


