# Supplementary material for: Polymorphisms of long non-coding RNA HOTAIR with breast cancer susceptibility and clinical outcomes for a southeast Chinese Han population
Source: Oncotarget. 2017 Dec 16;9(3):3677–89. doi: 10.18632/oncotarget.23343 (PMC5790492; doi:10.18632/oncotarget.23343)
Supplement: Supplementary file 1 [file oncotarget-09-3677-s001.pdf]

Polymorphisms of long non-coding RNA HOTAIR with breast cancer susceptibility and clinical outcomes for a southeast Chinese Han population

SUPPLEMENTARY MATERIALS

Supplementary Table 1: Stratified analysis on associations between rs4759314 polymorphism and breast cancer risk

Characteristics	rs4759314		*P*	OR (95% CI)[a]	*P*[b]
	Cases(AA/GA + GG)	Controls(AA/GA + GG)			
Age					0.606
≤40	223/53	237/44	0.391	1.22 (0.78–1.91)	
>40	578/115	580/109	0.752	1.05 (0.78–1.41)	
BMI					0.169
≤24	575/126	550/98	0.186	1.22 (0.91–1.64)	
>24	226/42	267/55	0.454	0.84 (0.53–1.33)	
Age at menarche					0.653
≤15	406/90	410/88	0.808	1.04 (0.75–1.45)	
>15	395/78	407/65	0.410	1.17 (0.81–1.70)	
Menopausal status					0.142
Premenopausal	510/110	534/90	0.160	1.25 (0.92–1.70)	
Postmenopausal	285/57	266/61	0.465	0.85 (0.57–1.30)	
Age at menopause					0.682
≤50	166/33	133/34	0.352	0.77 (0.44–1.34)	
>50	119/24	133/27	0.825	0.93 (0.50–1.74)	
Age at first live birth					0.148
≤25	426/98	546/97	0.212	1.22 (0.89–1.68)	
>25	344/63	235/52	0.367	0.83 (0.55–1.25)	
ER status					0.087
Positive	521/123		0.126	1.23 (0.94–1.61)	
Negative	280/45		0.249	0.81 (0.56–1.16)	
PR status					0.066
Positive	453/113		0.059	1.31 (0.99–1.72)	
Negative	348/55		0.177	0.79 (0.56–1.11)	

[a]Adjusted by age, BMI, age at menarche, menopausal status and family history of breast cancer where appropriate.
[b]*P* for heterogeneity test.

Supplementary Table 2: Stratified analysis of HOTAIR rs7958904 genotypes on DFS and OS of breast cancer patients

Variable	Disease free survival Genotypes (Relapse/Patients)					Overall survival Genotypes (Death/Patients)				
	GG	HR (95% CI)	GC + CC	HR (95% CI)[a*]	Log-rank *p*	GG	HR (95% CI)	GC + CC	HR (95% CI)[a*]	Log-rank *p*
Age										
≤40	46/129	1.00	62/147	1.16 (0.79–1.70)	0.460	25/129	1.00	41/147	1.35 (0.82–2.23)	0.236
>40	122/360	1.00	107/333	1.08 (0.83–1.40)	0.553	81/360	1.00	70/333	1.09 (0.79–1.50)	0.599
Tumor size										
≤2 cm	41/192	1.00	40/193	0.94 (0.61–1.46)	0.785	29/192	1.00	24/193	0.77 (0.45–1.33)	0.349
>2 cm	127/297	1.00	129/287	1.03 (0.81–1.32)	0.807	77/297	1.00	87/287	1.16 (0.85–1.58)	0.342
Lymph node involvement										
No	53/250	1.00	51/229	1.02 (0.70–1.51)	0.906	30/250	1.00	20/229	0.69 (0.39–1.22)	0.206
Yes	115/239	1.00	118/251	0.93 (0.72–1.20)	0.562	76/239	1.00	91/251	1.13 (0.83–1.53)	0.445
Molecular subtype										
Luminal type	97/313	1.00	94/331	0.86 (0.65–1.14)	0.299	57/313	1.00	52/331	0.83 (0.57–1.21)	0.324
HER-2 overexpression	34/83	1.00	31/66	1.23 (0.76–2.01)	0.401	23/83	1.00	23/66	1.37 (0.77–2.44)	0.287
TNBC	37/93	1.00	44/83	1.39 (0.90–2.16)	0.141	26/93	1.00	36/83	1.52 (0.91–2.53)	0.108

[a]Cox regression analyses for DFS and OS in breast cancer patients according to dominant model.
[*]Adjusted by age, tumor size, lymph node status, ER,PR and Her-2 status where appropriate.
